# diXa: a data infrastructure for chemical safety assessment

**DOI:** 10.1093/bioinformatics/btu827

**Published:** 2014-12-12

**Authors:** Diana M. Hendrickx, Hugo J.W.L. Aerts, Florian Caiment, Dominic Clark, Timothy M.D. Ebbels, Chris T. Evelo, Hans Gmuender, Dennie G.A.J. Hebels, Ralf Herwig, Jürgen Hescheler, Danyel G.J. Jennen, Marlon J.A. Jetten, Stathis Kanterakis, Hector C. Keun, Vera Matser, John P. Overington, Ekaterina Pilicheva, Ugis Sarkans, Marcelo P. Segura-Lepe, Isaia Sotiriadou, Timo Wittenberger, Clemens Wittwehr, Antonella Zanzi, Jos C.S. Kleinjans

**Affiliations:** ^1^Department of Toxicogenomics, School of Oncology and Developmental Biology (GROW), Maastricht University, 6200 MD Maastricht, The Netherlands, ^2^Dana-Farber Cancer Institute, Brigham and Women’s Hospital, Harvard Medical School, Boston, 02215, MA, USA, ^3^European Molecular Biology Laboratory – European Bioinformatics Institute, Wellcome Trust Genome Campus, Hinxton, Cambs CB10 1SD, UK, ^4^Computational and Systems Medicine, Department of Surgery and Cancer, Imperial College London, South Kensington, London SW7 2AZ, UK, ^5^Department of Bioinformatics – BiGCaT, Maastricht University, 6200 MD Maastricht, The Netherlands, ^6^Genedata AG, CH-4053 Basel, Switzerland, ^7^Department of Vertebrate Genomics, Max Planck Institute for Molecular Genetics, 14195 Berlin, Germany, ^8^Center of Physiology and Pathophysiology, Institute of Neurophysiology, University of Cologne, Cologne 50931, Germany and ^9^European Commission, Joint Research Centre, 21027 Ispra VA, Italy

## Abstract

**Motivation:** The field of toxicogenomics (the application of ‘-omics’ technologies to risk assessment of compound toxicities) has expanded in the last decade, partly driven by new legislation, aimed at reducing animal testing in chemical risk assessment but mainly as a result of a paradigm change in toxicology towards the use and integration of genome wide data. Many research groups worldwide have generated large amounts of such toxicogenomics data. However, there is no centralized repository for archiving and making these data and associated tools for their analysis easily available.

**Results:** The Data Infrastructure for Chemical Safety Assessment (diXa) is a robust and sustainable infrastructure storing toxicogenomics data. A central data warehouse is connected to a portal with links to chemical information and molecular and phenotype data. diXa is publicly available through a user-friendly web interface. New data can be readily deposited into diXa using guidelines and templates available online. Analysis descriptions and tools for interrogating the data are available via the diXa portal.

**Availability and implementation:** http://www.dixa-fp7.eu

**Contact:** d.hendrickx@maastrichtuniversity.nl; info@dixa-fp7.eu

**Supplementary information:** Supplementary data are available at *Bioinformatics* online.

## 1 Introduction

During the last decade, technology developments as well as new legislation, ethical considerations and concerns about the reliability and relevance of traditional animal experimentation for toxicity testing, have led to the expansion of the field of toxicogenomics (Hartung, 2009; Sycheva, *et al*., 2013). Many projects worldwide have generated large amounts of toxicogenomics data, but so far, there is no centralized repository collecting, curating and maintaining all these data. To make sure data are easily accessible and do not disappear over time, we developed the Data Infrastructure for Chemical Safety Assessment (diXa), a database and web interface providing access to toxicogenomics datasets and analysis.

While several toxicogenomics projects made their data already available via public databases (e.g. ArrayExpress, GEO, Expression Atlas), data from other projects are more difficult to access. Moreover, toxicogenomics data are generally deposited in isolation, not as structured sets. There are several reasons for this, among others: non comparable experimental designs, different technology platforms and different data (pre)processing steps. Furthermore, available metadata for public data sources are often insufficient for data reuse. diXa aims to overcome these drawbacks by defining standard workflows for data (pre)processing and standard formats for metadata annotation. These standards are applied to the diXa data through servicing. Moreover, diXa integrates information from toxicology, chemistry and human disease databases alongside the original data, helping interpretation of data analysis results and increasing the relevance for evaluating toxicity.

Combining data sets from different sources centrally can provide important information about experimental design and mechanistic interpretations. When all relevant data for a study are available in a public repository, a remaining challenge is to integrate these data in order to get a better understanding of the entire biological system (Gomez-Cabrero, *et al*., 2014; Schumacher, *et al*., 2014). Data from different platforms and different technologies are very heterogeneous in terms of experimental conditions, species, noise levels, time scales and linearity of response (Steinfath, *et al*., 2007). As a consequence, integrating data from different sources requires new data analysis methodologies (Gomez-Cabrero, *et al*., 2014).

Here we describe diXa, a database providing access to toxicogenomics data from different sources and data analysis tools.

## 2 Data infrastructure and access

**Fig. 1.**
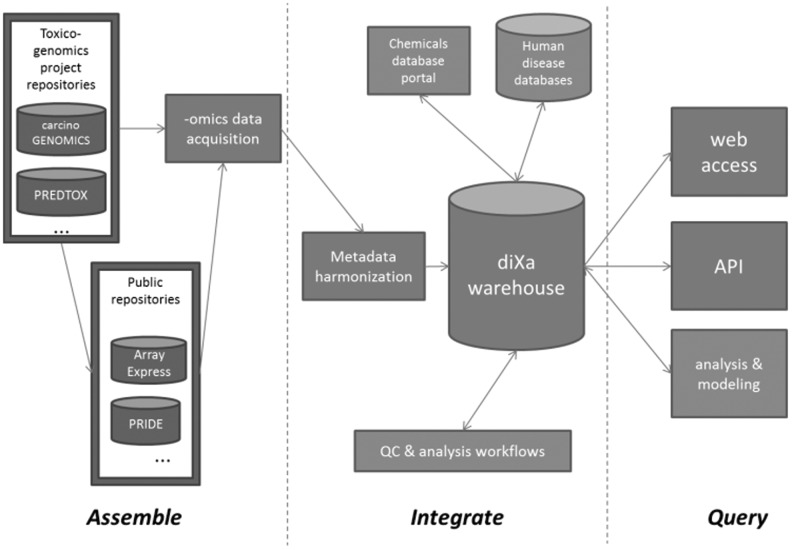
Overview of the diXa data infrastructure

diXa consists of a central warehouse containing data from toxicogenomics projects and other public repositories. The data warehouse is linked to a chemical portal as well as to a human disease database. An overview of diXa is presented in Figure 1.

### 2.1 Data sources

Currently, 34 studies involving 469 compounds are deposited in diXa, originating from various toxicogenomics projects (see Supplementary Table S1). The data have been generated through *in vitro* and *in vivo* rat and human transcriptomics, metabolomics and proteomics experiments. Additionally, diXa contains more recently measured Copy Number Variation and epigenetics data.

Data in diXa are described in ISA-Tab format (Rocca-Serra, *et al*., 2010; see Supplementary data, section ‘Uploading data’).

Understanding chemical, toxicity, and bioactivity properties of compounds under investigation is crucial in studying adverse outcomes (Stokstad, 2009). To provide direct access to curated public chemical databases, diXa is connected to the bioactivity database (ChEMBL; www.ebi.ac.uk/chembl/) and the JRC ChemAgora portal (chemagora.jrc.ec.europa.eu/). The ChemAgora portal provides direct access for each compound in the diXa data warehouse to chemical information available on third-party resources (see Supplementary Table S2): the portal, through an on-the-fly search, informs whether a compound has data in each of the external resources, and offers links leading to the exact third-party website pages where information about the compound can be found. Some third-party resources contain regulatory chemical information typically identified using the CAS Registry Number—this complements the use in the diXa data warehouse, of the standard InChIKey as core chemical structure identifier. Through ChemAgora a search is performed also in such third-party repositories, after the mapping of the InChiKey received from the diXa data warehouse into the corresponding CAS Registry Number.

### 2.2 Web interface

The diXa homepage (see Supplementary Fig. S1) provides ‘search’ and ‘browse’ sections allowing querying and browsing by studies, samples, compounds, analyses or diseases (see Supplementary Figs.S2–S11). The Experimental Factor Ontology (Malone, *et al*., 2010) is used to ensure that the contents can be also searched on synonyms and child terms. The ‘links’ section provides relevant information about diXa, among others on submitting data, training and novel analytical tools developed under diXa (Tools Catalogue).

To link studies to relevant chemical information, the ChemAgora portal provides options to perform searches for chemicals, based on InChIKeys (www.iupac.org), CAS Registry Numbers (www.cas.org), trivial names (including partial names), and structure.

### 2.3 Quality control, pre-processing and data analysis

Data deposited in diXa have been subject to quality control (QC), pre-processing and initial analyses (log2 ratios, differentially expressed genes) using pipelines implemented in Genedata Expressionist® (Hoefkens, *et al*., 2014). Researchers submitting data into diXa are requested to follow the guidelines mentioned above. Furthermore, there will be control on data completeness and standardization of meta-data through the use of ISA-Tab tools.

The algorithms used are described individually for each analysis and are published on the diXa homepage under “Analysis”. An overview of currently available analysis descriptions, together with their location on the diXa website is presented in Supplementary Table S4.

### 2.4 Applications

The accurate prediction of the toxicity of compounds remains a significant challenge. Availability of a centralized data warehouse allows combining data from different sources, including cross-omics analyses. Within diXa, it has been shown that combining data from *in vitro* studies on liver carcinogens with gene expression data from human liver cancers improved prediction of carcinogenicity (Caiment, *et al*., 2014). This also formed the basis of a promising approach for biomarker discovery for liver toxicity (Hebels, *et al*., 2014), where gene sets derived from different text mining and human liver ‘omics’ databases, were compared to determine the most promising gene lists for biomarker discovery. Furthermore, both studies showed that compound classifications based on *in vivo* data outperform classifications based on gene sets from the literature (‘expert knowledge’).

## 3 Current developments

diXa is a sustainable data-infrastructure. It will be updated for storing more data types and classes, including next generation sequencing and methylation data. Furthermore, new tools for integrated statistical analysis will be developed and added to diXa. diXa has already been adopted as the informatics framework for the EU FP7 HeCaTos project (http://www.hecatos.eu/).

The ChemAgora portal is also a long-term strategic development, to which the European Commission’s Joint Research Centre is fully committed. ChemAgora has already caught the attention of other initiatives, e.g. IPCheM (http://ipchem.jrc.ec.europa.eu/), a European Commission project, which will take advantage of the search service provided by ChemAgora.

## 4 Conclusion

diXa is a stable and long-term data repository providing free public access to toxicogenomics data. A web interface with several query tools was implemented, allowing users to search and browse diXa. We expect that the extensive use of structured metadata will have large impact on implementation, in particular by allowing flexible application in future use cases.

## Supplementary Material

Supplementary Data
