# Supplementary material for: diXa: a data infrastructure for chemical safety assessment
Source: Bioinformatics. 2014 Dec 12;31(9):1505–7. doi: 10.1093/bioinformatics/btu827 (PMC4410652; doi:10.1093/bioinformatics/btu827)
Supplement: Supplementary Data [file supp_31_9_1505__index.html]

diXa: a Data Infrastructure for Chemical Safety Assessment — diXa: a data infrastructure for chemical safety assessment — diXa: a data infrastructure for chemical safety assessment — Supplementary Data 

# diXa: a data infrastructure for chemical safety assessment

## Supplementary Data

files

**Files in this Data Supplement:**

- Supplementary Data - pdf file
