# Supplementary material for: diXa: a data infrastructure for chemical safety assessment
Source: Bioinformatics. 2014 Dec 12;31(9):1505–7. doi: 10.1093/bioinformatics/btu827 (PMC4410652; doi:10.1093/bioinformatics/btu827)
Supplement: Supplementary Data [file supp_btu827_Supplementary_data_rev_final.pdf]

# Supplementary data for "diXa: a Data Infrastructure for Chemical Safety Assessment"

Diana M. Hendrickx[1,*], Hugo J.W.L. Aerts[2], Florian Caiment[1], Dominic Clark[3], Timothy M.D. Ebbels[4], Chris T. Evelo[5], Hans Gmuender[6], Dennie G.A.J. Hebels[1], Ralf Herwig[7], Jürgen Hescheler[8], Danyel G.J. Jennen[1], Marlon J.A. Jetten[1], Stathis Kanterakis[3], Hector C. Keun[4], Vera Matser[3], John P. Overington[3], Ekaterina Pilicheva[3], Ugis Sarkans[3], Marcelo P. Segura-Lepe[4], Isaia Sotiriadou[8], Timo Wittenberger[6], Clemens Wittwehr[9], Antonella Zanzi[9], and Jos C.S. Kleinjans[1]

[1]Department of Toxicogenomics, School of Oncology and Developmental Biology (GROW), Maastricht University, 6200 MD Maastricht, The Netherlands. [2] Dana-Farber Cancer Institute, Brigham and Women's Hospital, Harvard Medical School, Boston, 02215, MA, USA. [3]European Molecular Biology Laboratory – European Bioinformatics Institute, Wellcome Trust Genome Campus, Hinxton, Cambs, CB10 1SD, United Kingdom. [4] Computational and Systems Medicine, Department of Surgery and Cancer, Imperial College London, South Kensington, London SW7 2AZ, UK. [5] Department of Bioinformatics – BiGCaT Bioinformatics and Systems Biology Research Group, Maastricht University, 6200 MD Maastricht, The Netherlands. [6]Genedata AG, CH-4053 Basel, Switzerland. [7]Department of Vertebrate Genomics, Max Planck Institute for Molecular Genetics, 14195 Berlin, Germany. [8]Center of Physiology and Pathophysiology, Institute of Neurophysiology, University of Cologne, Cologne 50931, Germany. [9] European Commission , Joint Research Centre, Via E. Fermi 2749, 21027 Ispra VA, Italy.

**Table S1** Overview of projects participating in diXa

| Project | Description | Weblink | Organism(s) | Organ(s) / tissue(s) | Measurement type | Technology type |
|---|---|---|---|---|---|---|
| New Generis<br>(1 study) | Links between exposure to environmental/food carcinogens during pregnancy and childhood cancer | http://www.newgeneris.org/ | Homo Sapiens | Human PBMC | Transcription profiling | DNA microarray (Agilent) |
| Carcinogenomics<br>(3 studies) | High throughput genomics-based tests for assessing genotoxic and carcinogenic properties of chemical compounds *in vitro* | http://www.carcinogenomics.eu/ | Homo Sapiens<br>Rattus norvegicus | Cell lines: liver (human and rat), kidney (human and rat), lung (human) | Metabolite profiling (only liver and kidney)<br>Transcription profiling (liver, kidney, lung) | NMR spectroscopy (Bruker)<br>DNA microarray (Affymetrix) |
| Envirogenomarkers<br>(2 studies) | Role of environmental agents in human disease | http://www.envirogenomarkers.net/ | Homo Sapiens | Blood specimen | Transcription profiling | DNA microarray (Agilent) |
| InnoMed – PredTox<br>(16 studies) | Pre-clinical safety evaluation of compounds | https://www.genedata.com/lp/innomed-predtox.html | Rattus norvegicus | Urine, liver, kidney, serum, blood, plasma | Transcription profiling<br>Metabolite profiling<br>Protein expression profiling<br>Hematology<br>Histology<br>Clinical chemistry analysis | DNA microarray (Affymetrix GeneChip)<br>Mass spectrometry (LC/MS)<br>NMR spectroscopy (1D NMR)<br>Mass spectrometry (SELDI-TOF-MS, 2D-PAGE/MALDI-TOF/MS, 2D-DIGE/MALDI-TOF-MS) |
| Esnats<br>(2 studies) | Embryonic stem cell-based alternative novel testing stategies | http://www.esnats.eu/ | Homo Sapiens | Embryonic stem cells, skin-derived stem cells | Transcription profiling | DNA microarray (Affymetrix) |
| Predictomics<br>(1 study) | Short-term in vitro assays for long-term toxicity | http://www.ist-world.org/ProjectDetails.aspx?ProjectId=db2e81aa1bf442559aa3d2eea5b20c4f&SourceDatabaseId=7cff9226e582440894200b751bab883f | Homo Sapiens | Liver cell lines | Transcription profiling | DNA microarray (Affymetrix) |
| Open TG-GATEs<br>(4 studies) | Toxicity information for 150 compounds on rat in vivo, liver and kidney cells, and human hepatocytes | http://toxico.nibio.go.jp/english/index.html | Homo Sapiens<br>Rattus norvegicus | Liver cell lines (rat and human)<br>Liver (only rat)<br>Kidney (only rat) | Transcription profiling | DNA microarray (Affymetrix) |
| DrugMatrix<br>(5 studies) | Toxicogenomic profiles for 638 different compounds | https://ntp.niehs.nih.gov/drugmatrix/index.html | Rattus norvegicus | Kidney, liver, heart, liver cell lines, skeletal muscle tissue | Transcription profiling | DNA microarray (Affymetrix) |

**Table S2** Current external sources linked to the diXa Data Warehouse through the ChemAgora Portal.

| Database | Weblink | Reference |
|---|---|---|
| ChEMBL | https://www.ebi.ac.uk/chembl/ | (Warr, 2009) |
| ChEBI | http://www.ebi.ac.uk/chebi/ | (Degtyarenko, et al., 2008) |
| ChemSpider | http://www.chemspider.com/ | (Pence and Williams, 2010) |
| PubChem | https://pubchem.ncbi.nlm.nih.gov/ | (Wang, et al., 2009) |
| eChemPortal | http://www.echemportal.org | (Wittwehr, 2011) |
| ConsensusPathDB | http://cpdb.molgen.mpg.de/ | (Kamburov, et al., 2013) |
| CTD | http://ctdbase.org/ | (Davis, et al., 2013) |
| DrugBank | http://www.drugbank.ca/ | (Law, et al., 2014) |
| ToxNet | http://toxnet.nlm.nih.gov/ | (Wexler, 2001) |
| CommonChemistry | http://www.commonchemistry.org/ | - |
| AOP-Wiki | https://aopkb.org/aopwiki/ | - |
| CheList | http://chelist.jrc.ec.europa.eu/ | - |

## Uploading data

New data are added to the diXa repository in ISA-Tab format (Rocca-Serra, et al., 2010) (isatab.sourceforge.net/tools.html). ISA-Tab is a metadata format that describes investigations, studies and assays, and ties together this information using links to the experimental data. The data catalogues used by the diXa studies are available in ArrayExpress (Rustici, et al., 2013).
The diXa ISA template can be downloaded from bitbucket.org/kanterae/isaconfig-dixa/src. The template has additional fields related to toxicology, such as ‘dose’ and ‘treatment’ and uses several ontologies (see Supplementary Table S3).

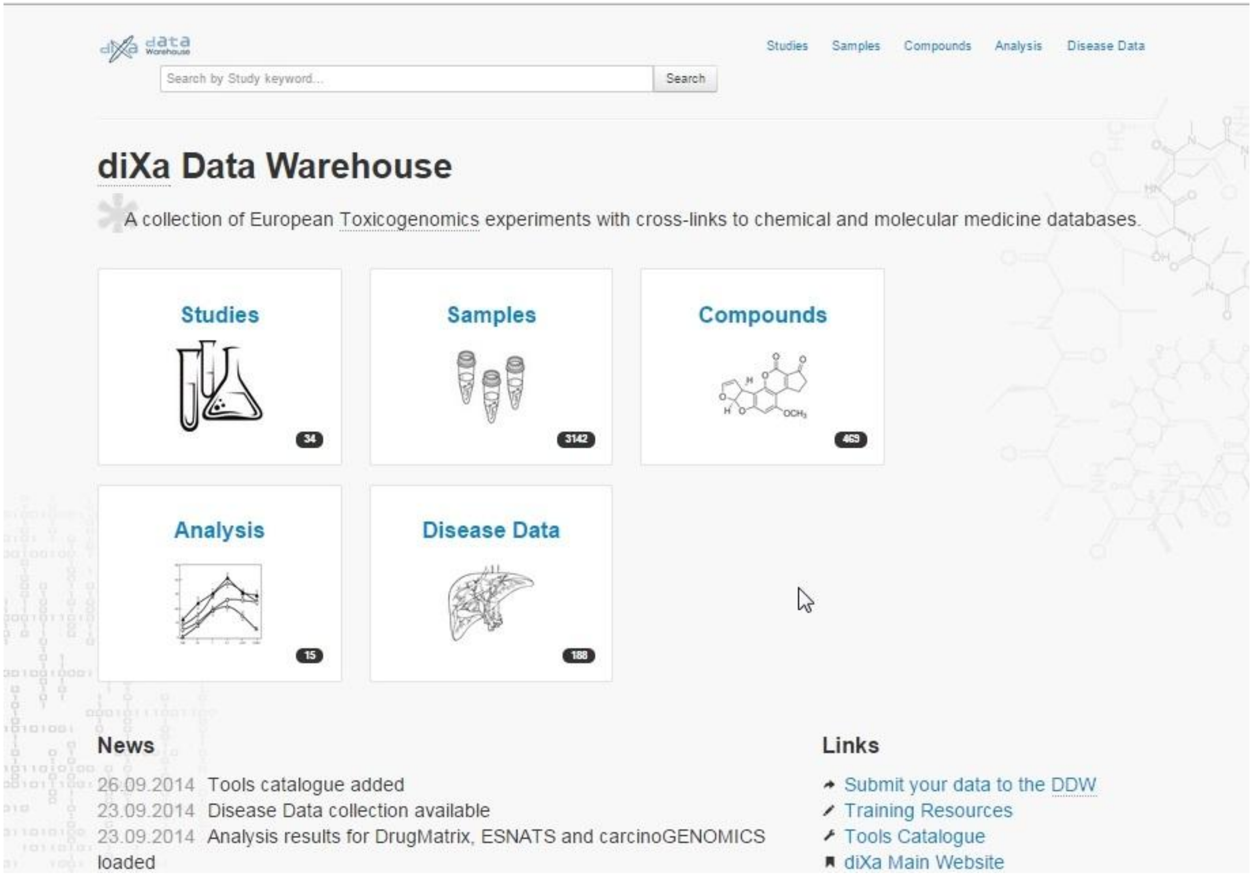


**Fig. S1** diXa Data Warehouse homepage

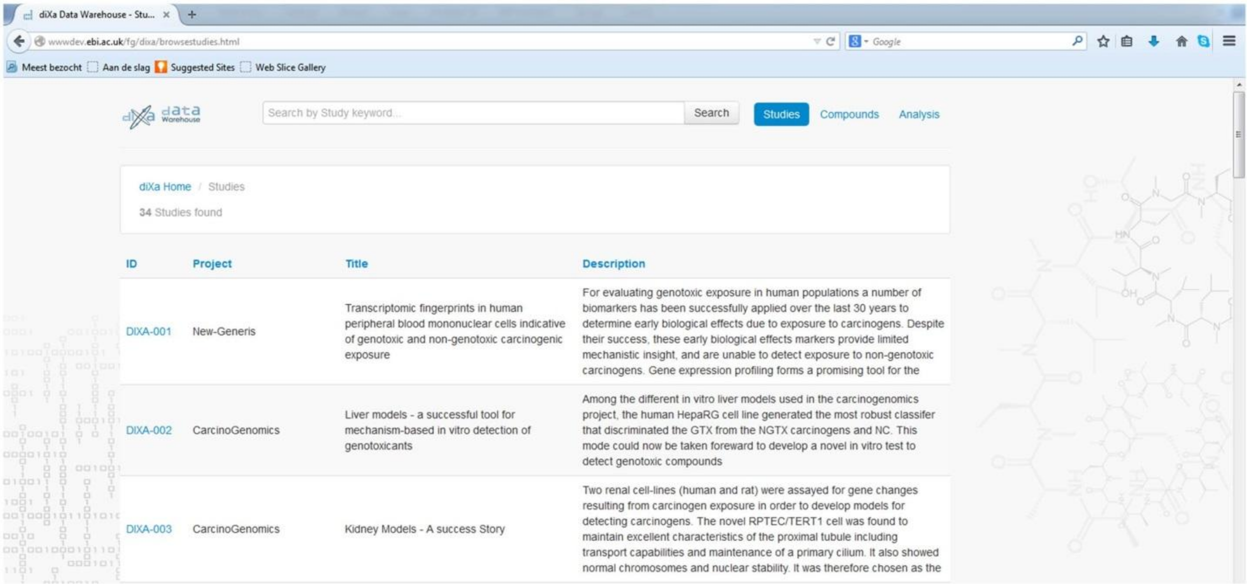


**Fig. S2** The Study list view shows all studies matching a search query or the complete list of studies when browsing.

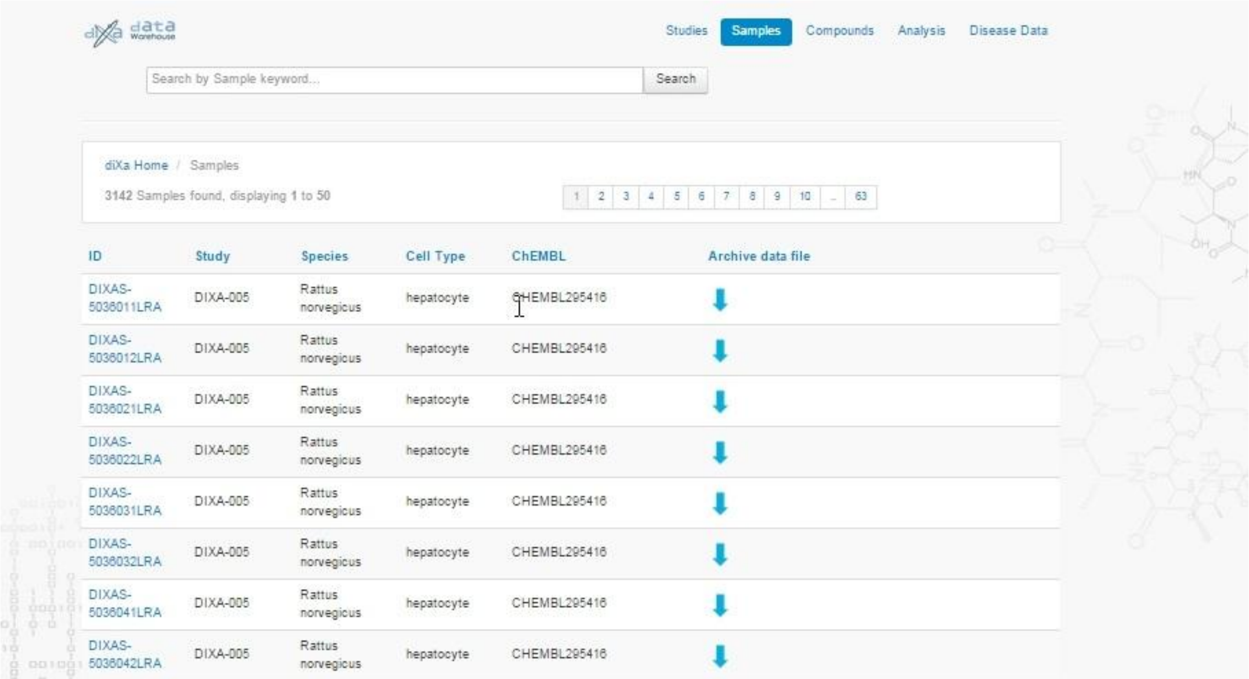


**Fig. S3** The Sample list view shows all samples matching a search query or the complete list of samples when browsing.

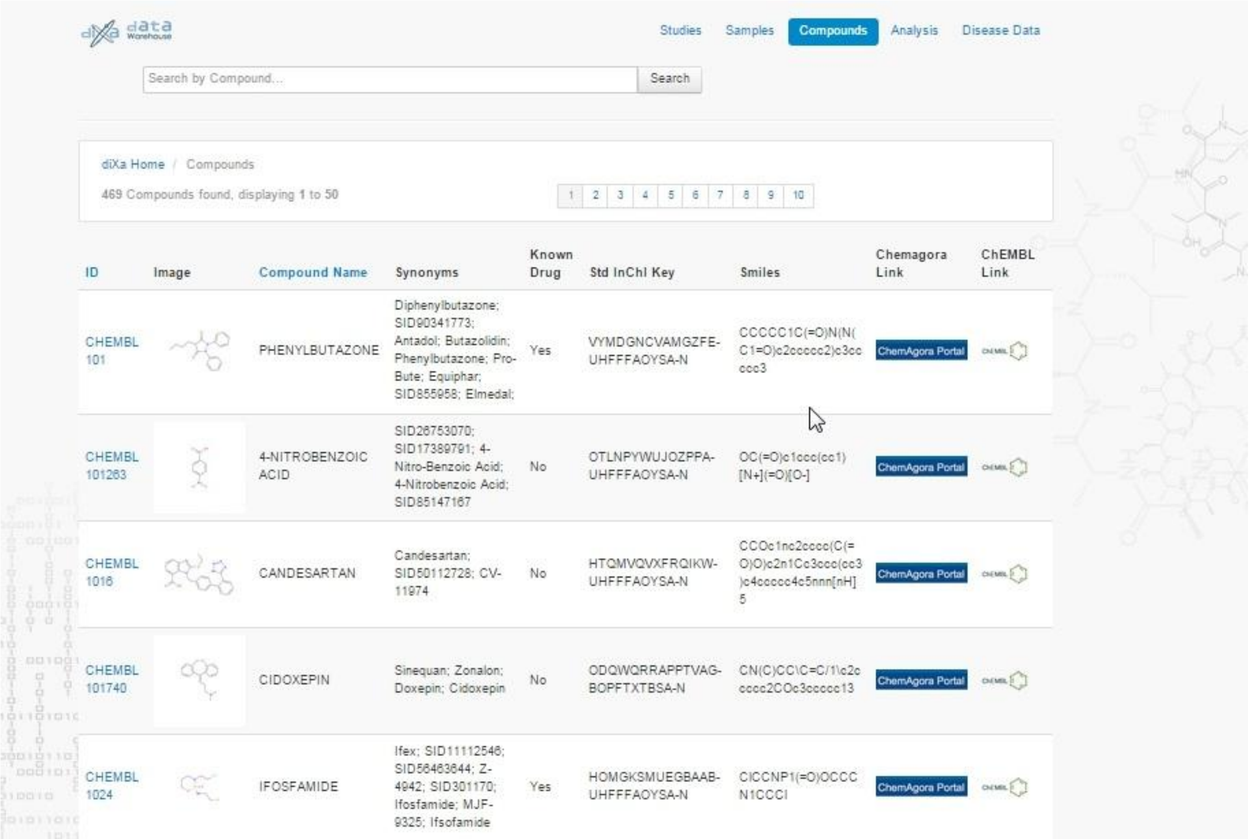


**Fig. S4** The Compound list view shows all compounds matching a search query or the complete list of compounds when browsing.

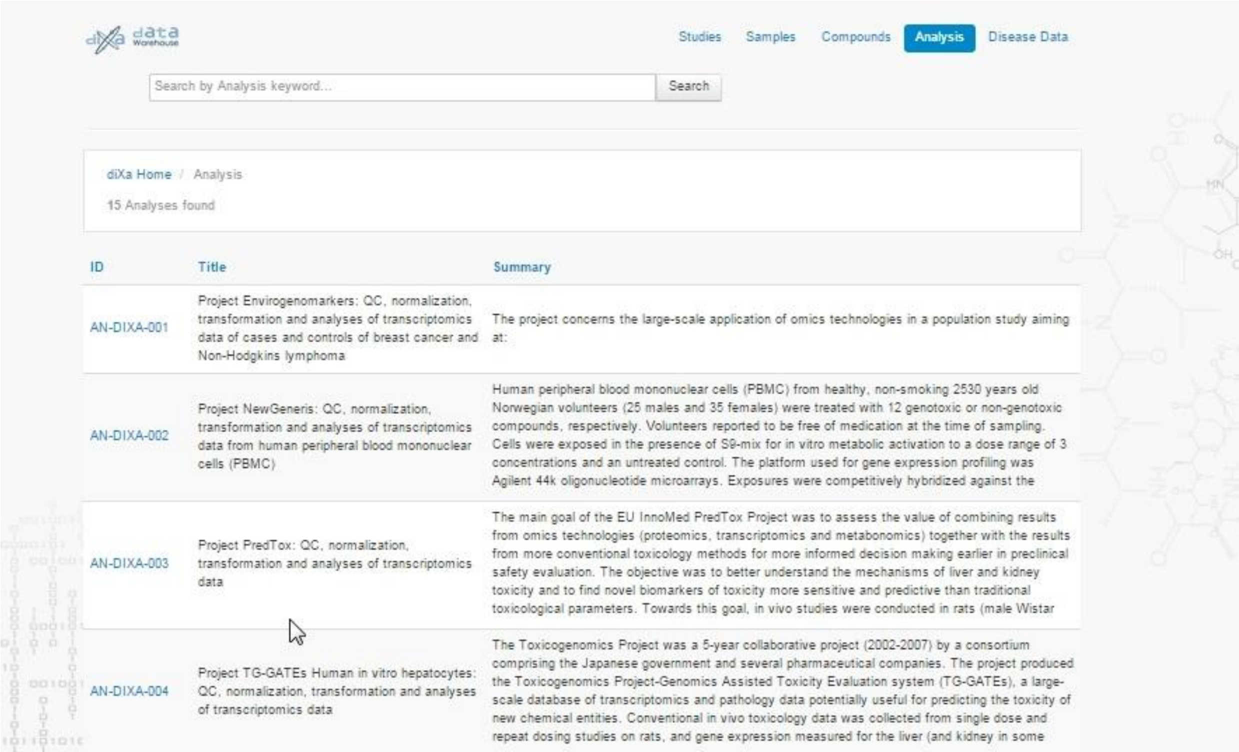


**Fig. S5** The Analysis list view shows all analyses matching a search query or the complete list of analyses when browsing.

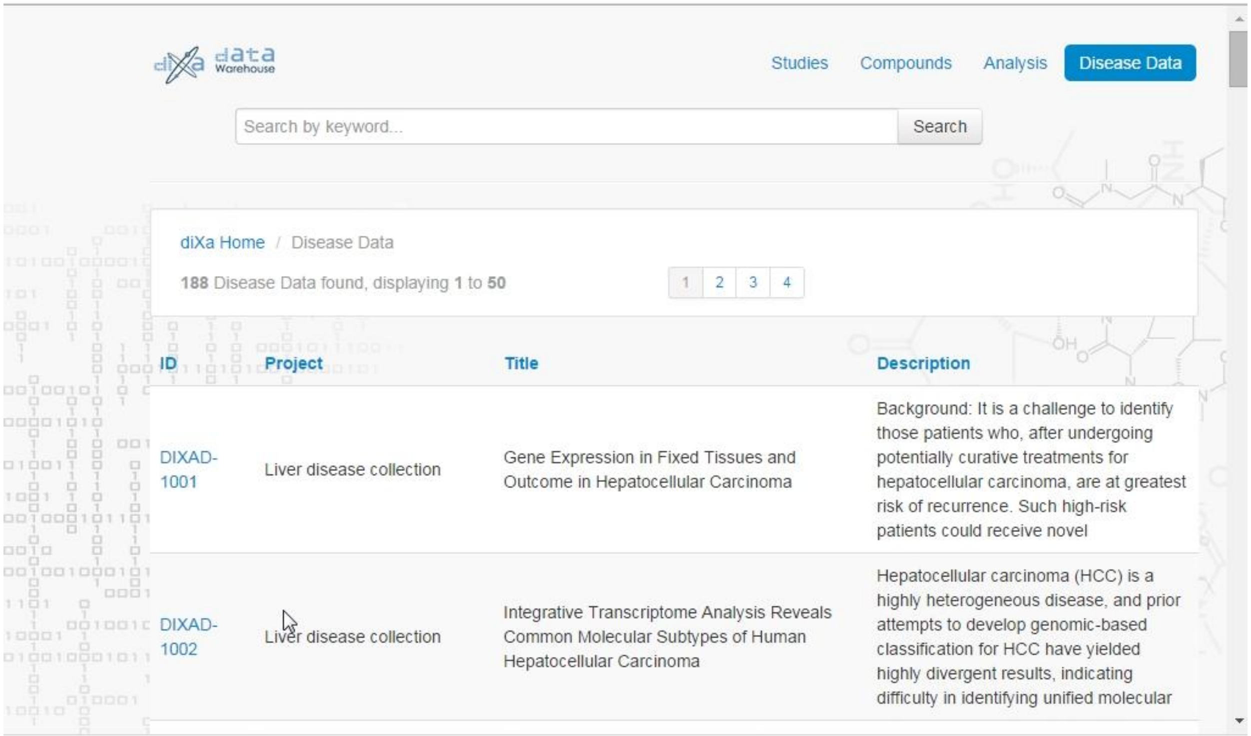


**Fig. S6** The Disease list view shows all diseases matching a search query or the complete list of diseases when browsing.

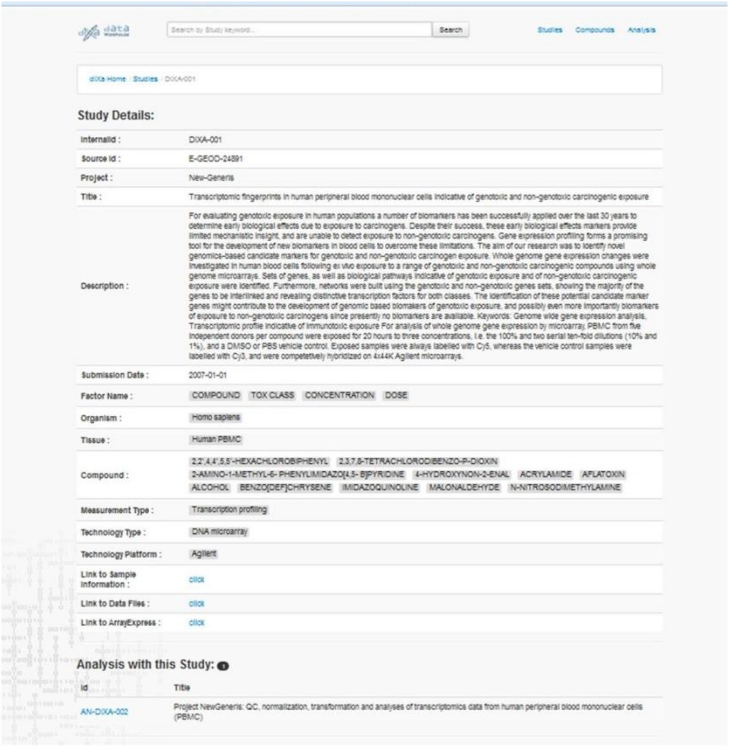


**Fig. S7** The Study detail view shows additional information on a selected study. Furthermore, the study detail view provides links to the repositories were the experimental data are stored and links to detailed information on the compounds in the study.

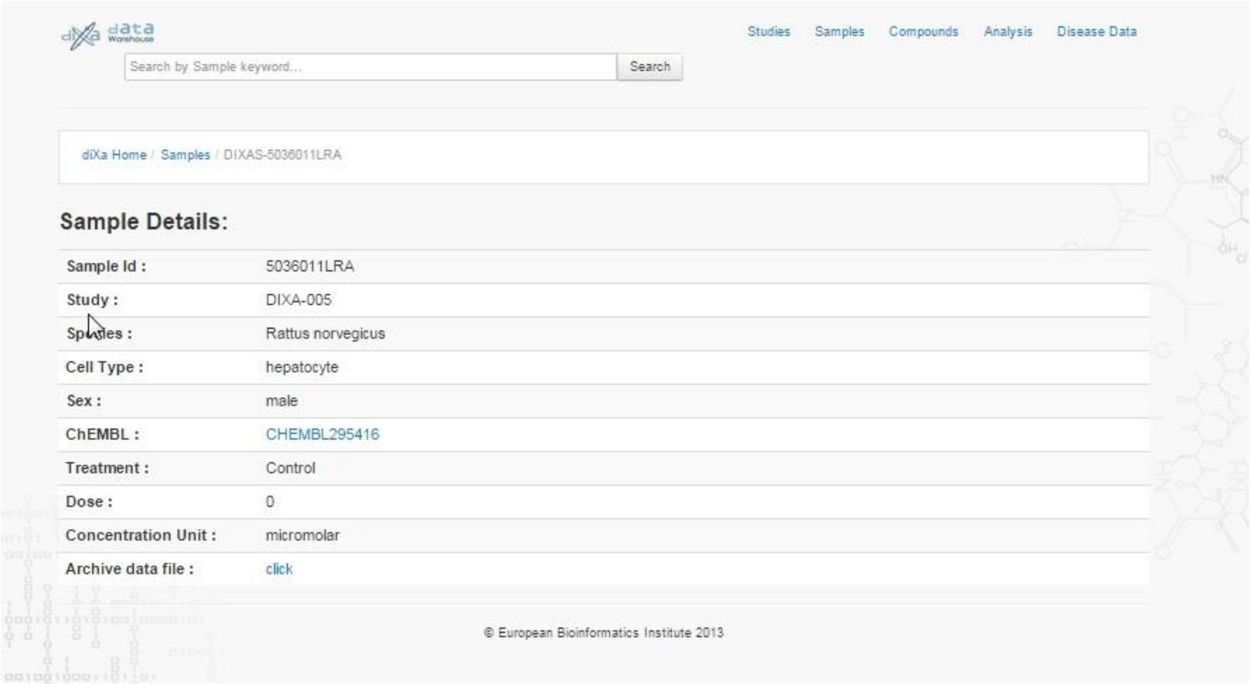


**Fig. S8** The Sample detail view shows additional information on a selected sample. Furthermore, the sample detail view provides links to ChEMBL and to the data file for the selected sample.

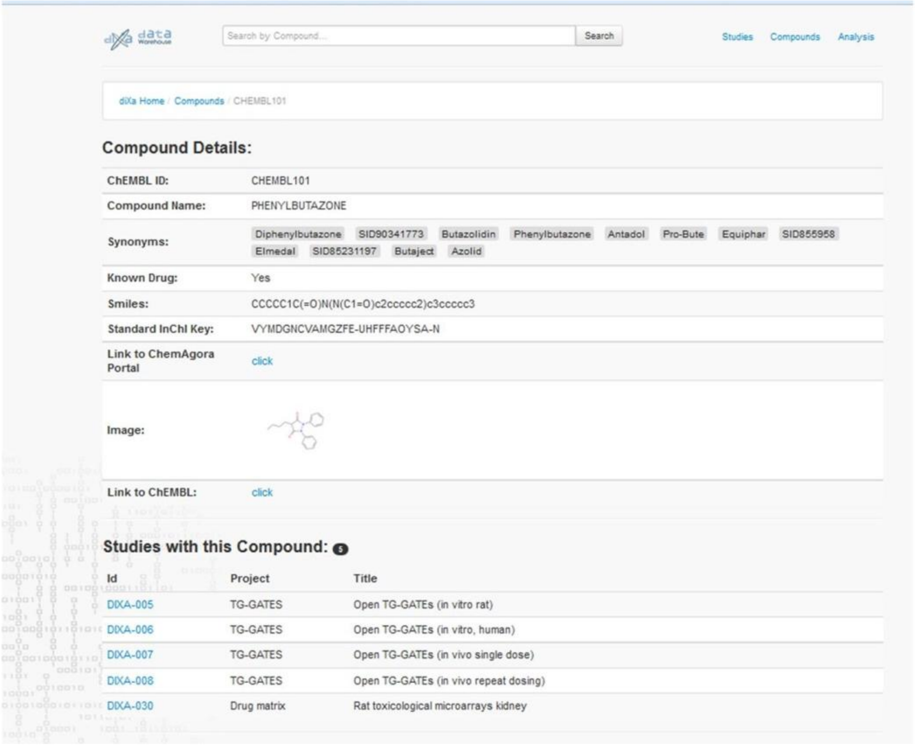


**Fig. S9** The Compound detail view shows additional information on a selected compound. Moreover, the compound detail view links to ChemAgora Portal, ChEMBL and studies with the selected compound.

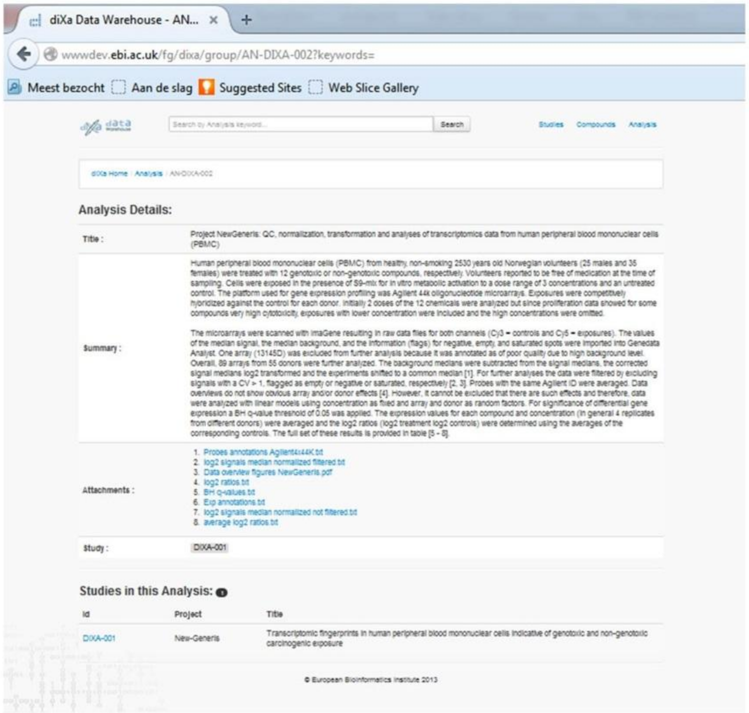


**Fig. S10** The Analysis detail view shows additional information on a selected analysis. Furthermore, the analysis detail view provides access to the files containing the results and studies related to a selected analysis.

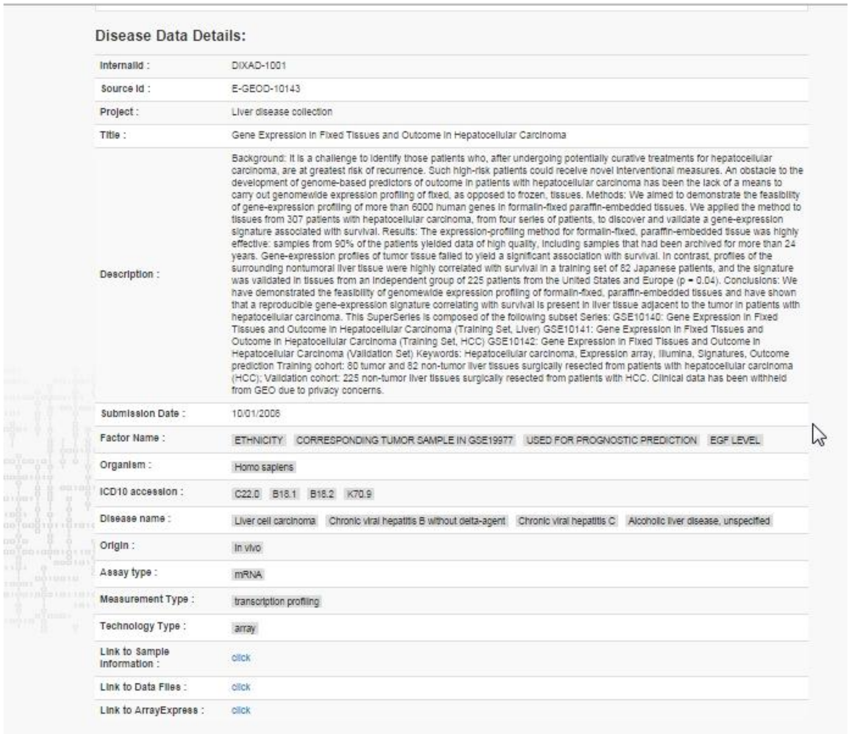

**Disease Data Details:**

| | |
|---|---|
| InternalId : | DIXAD-1001 |
| Source Id : | E-GEOD-10143 |
| Project : | Liver disease collection |
| Title : | Gene Expression in Fixed Tissues and Outcome in Hepatocellular Carcinoma |
| Description : | Background: It is a challenge to identify those patients who, after undergoing potentially curative treatments for hepatocellular carcinoma, are at greatest risk of recurrence. Such high-risk patients could receive novel interventional measures. An obstacle to the development of genome-based predictors of outcome in patients with hepatocellular carcinoma has been the lack of a means to carry out genomewide expression profiling of fixed, as opposed to frozen, tissues. Methods: We aimed to demonstrate the feasiblity of gene-expression profiling of more than 6000 human genes in formalin-fixed paraffin-embedded tissues. We applied the method to tissues from 307 patients with hepatocellular carcinoma, from four series of patients, to discover and validate a gene-expression signature associated with survival. Results: The expression-profiling method for formalin-fixed, paraffin-embedded tissue was highly effective: samples from 90% of the patients yielded data of high quality, including samples that had been archived for more than 24 years. Gene-expression profiles of tumor tissue failed to yield a significant association with survival. In contrast, profiles of the surrounding nontumoral liver tissue were highly correlated with survival in a training set of 82 Japanese patients, and the signature was validated in tissues from an independent group of 225 patients from the United States and Europe (p = 0.04). Conclusions: We have demonstrated the feasiblity of genomewide expression profiling of formalin-fixed, paraffin-embedded tissues and have shown that a reproducible gene-expression signature correlating with survival is present in liver tissue adjacent to the tumor in patients with hepatocellular carcinoma. This SuperSeries is composed of the following subset Series: GSE10140: Gene Expression in Fixed Tissues and Outcome in Hepatocellular Carcinoma (Training Set, Liver) GSE10141: Gene Expression in Fixed Tissues and Outcome in Hepatocellular Carcinoma (Training Set, HCC) GSE10142: Gene Expression in Fixed Tissues and Outcome in Hepatocellular Carcinoma (Validation Set) Keywords: Hepatocellular carcinoma, Expression array, Illumina, Signatures, Outcome prediction Training cohort: 80 tumor and 82 non-tumor liver tissues surgically resected from patients with hepatocellular carcinoma (HCC); Validation cohort: 225 non-tumor liver tissues surgically resected from patients with HCC. Clinical data has been withheld from GEO due to privacy concerns. |
| Submission Date : | 10/01/2008 |
| Factor Name : | ETHNICITY, CORRESPONDING TUMOR SAMPLE IN GSE19977, USED FOR PROGNOSTIC PREDICTION, EGF LEVEL |
| Organism : | Homo sapiens |
| ICD10 accession : | C22.0, B18.1, B18.2, K70.9 |
| Disease name : | Liver cell carcinoma, Chronic viral hepatitis B without delta-agent, Chronic viral hepatitis C, Alcoholic liver disease, unspecified |
| Origin : | in vivo |
| Assay type : | mRNA |
| Measurement Type : | transcription profiling |
| Technology Type : | array |
| Link to Sample Information : | click |
| Link to Data Files : | click |
| Link to ArrayExpress : | click |

**Fig. S11** The Disease detail view shows additional information on a selected disease. Furthermore, the disease detail view provides links to the repositories were the experimental data are stored.

**Table S3** Overview of ontologies used by diXa ISA template.

| Ontology | Weblink |
|---|---|
| NCBI Organismal Classification (NCBITAXON) | http://bioportal.bioontology.org/ontologies/NCBITAXON |
| Units of Measurements Ontology (UO) | http://bioportal.bioontology.org/ontologies/UO |
| Ontology for Biomedical Investigations (OBI) | http://bioportal.bioontology.org/ontologies/OBI |
| Chemical Entities of Biological Interest (ChEBI) | http://bioportal.bioontology.org/ontologies/CHEBI |
| NCI Thesaurus (NCIT) | http://bioportal.bioontology.org/ontologies/NCIT |

**Table S4** Overview of currently available analysis descriptions, together with their location on the diXa website.

| Analysis description | Weblink |
|---|---|
| Project Envirogenomarkers: QC, normalization, transformation and analyses of transcriptomics data of cases and controls of breast cancer and Non-Hodgkins lymphoma | http://wwwdev.ebi.ac.uk/fg/dixa/group/AN-DIXA-001 |
| Project NewGeneris: QC, normalization, transformation and analyses of transcriptomics data from human peripheral blood mononuclear cells (PBMC) | http://wwwdev.ebi.ac.uk/fg/dixa/group/AN-DIXA-002 |
| Project PredTox: QC, normalization, transformation and analyses of transcriptomics data | http://wwwdev.ebi.ac.uk/fg/dixa/group/AN-DIXA-003 |
| Project TG-GATEs Human in vitro hepatocytes: QC, normalization, transformation and analyses of transcriptomics data | http://wwwdev.ebi.ac.uk/fg/dixa/group/AN-DIXA-004 |
| Project TG-GATEs Rat in vitro hepatocytes: QC, normalization, transformation and analyses of transcriptomics data | http://wwwdev.ebi.ac.uk/fg/dixa/group/AN-DIXA-005 |
| Project TG-GATEs Rat in vivo Kidney: QC, normalization, transformation and analyses of transcriptomics data | http://wwwdev.ebi.ac.uk/fg/dixa/group/AN-DIXA-006 |
| Project TG-GATEs Rat in vivo Liver: QC, normalization, transformation and analyses of transcriptomics data | http://wwwdev.ebi.ac.uk/fg/dixa/group/AN-DIXA-007 |
| Project Predictomics: QC, normalization, transformation and analyses of transcriptomics data of test substances (hepatotoxins) in human primary hepatocytes and HepG2 cells | http://wwwdev.ebi.ac.uk/fg/dixa/group/AN-DIXA-008 |
| Project DrugMatrix: QC, normalization, transformation and analyses of transcriptomics data from rat kidney, heart, liver, skeletal muscle tissue, and rat hepatocytes | http://wwwdev.ebi.ac.uk/fg/dixa/group/AN-DIXA-009 |
| Project ESNATS: QC, normalization, transformation and analyses of transcriptomics data from human embryonic stem cells | http://wwwdev.ebi.ac.uk/fg/dixa/group/AN-DIXA-010 |
| Project PredTox: QC, normalization, transformation and analyses of serum metabolites by LC/MS | http://wwwdev.ebi.ac.uk/fg/dixa/group/AN-DIXA-011 |
| Project carcinoGENOMICS: QC, normalization, transformation and analyses of transcriptomics data of the kidney (RPTEC/TERT1) and liver (HepaRG) cell lines | http://wwwdev.ebi.ac.uk/fg/dixa/group/AN-DIXA-012 |
| Project carcinoGENOMICS: QC, normalization, transformation and analyses of transcriptomics data of the liver cell line HepG2 | http://wwwdev.ebi.ac.uk/fg/dixa/group/AN-DIXA-013 |
| Project carcinoGENOMICS: QC, normalization, transformation and analyses of transcriptomics data of Human Embryonic Stem Cell Derived Hepatocyte-Like Cells (hESC DE-Hep) | http://wwwdev.ebi.ac.uk/fg/dixa/group/AN-DIXA-014 |
| Project carcinoGENOMICS: QC, normalization, transformation and analyses of transcriptomics data in a lung epithelial cell-based test system | http://wwwdev.ebi.ac.uk/fg/dixa/group/AN-DIXA-015 |

## References for Table S2 and the text under Table S2.

Davis, A.P., *et al.* (2013) The Comparative Toxicogenomics Database: update 2013, *Nucleic Acids Res.*, **41**, D1104-1114.

Degtyarenko, K., *et al.* (2008) ChEBI: a database and ontology for chemical entities of biological interest, *Nucleic Acids Res.*, **36**, D344-D350.

Kamburov, A., *et al.* (2013) The ConsensusPathDB interaction database: 2013 update, *Nucleic Acids Res.*, **41**, D793-D800.

Law, V., *et al.* (2014) DrugBank 4.0: shedding new light on drug metabolism, *Nucleic acids research*, **42**, D1091-1097.

Pence, H.E. and Williams, A. (2010) ChemSpider: an online chemical information resource, *J. Chem. Educ.*, **87**, 1123-1124.

Rocca-Serra, P., *et al.* (2010) ISA software suite: supporting standards-compliant experimental annotation and enabling curation at the community level, *Bioinformatics*, **26**, 2354-2356.

Rustici, G., *et al.* (2013) ArrayExpress update - trends in database growth and links to data analysis tools, *Nucl. Acids Res*, **41**, D987-D990.

Wang, Y., *et al.* (2009) PubChem: a public information system for analyzing bioactivities of small molecules, *Nucleic acids research*, **37**, W623-633.

Warr, W. (2009) ChEMBL. An interview with John Overington, team leader, chemogenomics at the European Bioinformatics Institute Outstation of the European Molecular Biology Laboratory (EMBL-EBI), *J Comput Aid Mol Des*, **23**, 195-198.

Wexler, P. (2001) TOXNET: an evolving web resource for toxicology and environmental health information, *Toxicology*, **157**, 3-10.

Wittwehr, C. (2011) Neuer Zugang zu Chemikalien-Daten. eChemPortal, *Chemie in unserer Zeit*, **45**, 122-125.
